# Clinical and genetic characteristics of chinese patients with Birt-Hogg-Dubé syndrome

**DOI:** 10.1186/s13023-017-0656-7

**Published:** 2017-05-30

**Authors:** Yaping Liu, Zhiyan Xu, Ruie Feng, Yongzhong Zhan, Jun Wang, Guozhen Li, Xue Li, Weihong Zhang, Xiaowen Hu, Xinlun Tian, Kai-Feng Xu, Xue Zhang

**Affiliations:** 1 McKusick-Zhang Center for Genetic Medicine, State Key Laboratory of Medical Molecular Biology, Institute of Basic Medical Sciences, Chinese Academy of Medical Sciences and Peking Union Medical College, Beijing, 100005 China; 2 Department of Internal Medicine, Peking Union Medical College Hospital, Beijing, China; 3 Department of Pathology, Peking Union Medical College Hospital, Beijing, China; 4 Department of Respiratory Medicine, Chinese Academy of Medical Sciences, Peking Union Medical College Hospital, Beijing, 100730 China; 5 Department of Respiratory Medicine, Nanfang Hospital, Southern Medical University, Guangzhou, China; 6 Department of Radiology, Peking Union Medical College Hospital, Beijing, China; 7 Department of Respiratory Medicine, Anhui Provincial Hospital Affiliated to Anhui Medical University, Hefei, China

**Keywords:** Birt-Hogg-Dubé syndrome, *FLCN*, Mutation spectrum

## Abstract

**Background:**

Birt-Hogg-Dubé syndrome (BHD) is an autosomal dominant disorder, the main manifestations of which are fibrofolliculomas, renal tumors, pulmonary cysts and recurrent pneumothorax. The known causative gene for BHD syndrome is the folliculin (*FLCN*) gene on chromosome 17p11.2. Studies of the *FLCN* mutation for BHD syndrome are less prevalent in Chinese populations than in Caucasian populations. Our study aims to investigate the genotype spectrum in a group of Chinese patients with BHD.

**Methods:**

We enrolled 51 patients with symptoms highly suggestive of BHD from January 2014 to February 2017. The *FLCN* gene was examined using PCR and *Sanger* sequencing in every patient, for those whose *Sanger* sequencing showed negative mutation results, multiplex ligation-dependent probe amplification (MLPA) testing was conducted to detect any losses of large segments.

**Main results:**

Among the 51 patients, 27 had *FLCN* germline mutations. In total, 20 mutations were identified: 14 were novel mutations, including 3 splice acceptor site mutations, 2 different deletions, 6 nonsense mutations, 1 missense mutation, 1 small insertion, and 1 deletion of the whole exon 8.

**Conclusions:**

We found a similar genotype spectrum but different mutant loci in Chinese patients with BHD compared with European and American patients, thus providing stronger evidence for the clinical molecular diagnosis of BHD in China. It suggests that mutation analysis of the *FLCN* gene should be systematically conducted in patients with cystic lung diseases.

**Electronic supplementary material:**

The online version of this article (doi:10.1186/s13023-017-0656-7) contains supplementary material, which is available to authorized users.

## Background

Birt-Hogg-Dubé syndrome (BHD, OMIM #135150) is a rare autosomal dominant disorder, the main symptoms of which are multiple pulmonary cysts followed by recurrent pneumothorax, fibrofolliculomas, and renal cell carcinomas. These three symptoms appear separately [1, 2]. Lung-related symptoms are often the earliest phenotypical manifestations to appear, but most patients are asymptomatic [3, 4]. The pulmonary manifestations of BHD occasionally need to be distinguished from other conditions associated with diffuse cysts lung diseases (DCLD), such as lymphangioleiomyomatosis, Langerhans cell histiocytosis, lymphocytic interstitial and pneumonitis [5]. Lack of a comprehensive understanding of BHD often leads to a high misdiagnosis rate.

The gene responsible for BHD syndrome, the folliculin (*FLCN*) gene on chromosome 17p11.2, is a tumor suppressor gene that was first reported in 2002 [6] and is known to be involved in the signaling of mammalian target of rapamycin (mTOR) [7]. The *FLCN* gene consists of 14 exons encoding a 579-amino-acid-long protein, folliculin [8]. Disease-causing mutations in the *FLCN* gene, including insertions, deletions, missense and nonsense mutations, were found over the entire gene. Multiple in vitro studies focused on FLCN functions suggested that the activation of the AKT-mTOR pathway and increased activity of basic-helix-loop-helix transcription factor TFE3 were related to *FLCN*-deficient mouse cell lines [9–11]. To date, 149 unique *FLCN* germline mutations have been identified in BHD patients and catalogued in the Leiden Open Variation Database. Since Nickerson et al. first described BHD syndrome in 2002 [6], studies of *FLCN* mutations and related manifestations have been popular in Europe and the United States. Analysis of Caucasian data demonstrated that the frequency of 1-bp deletion or insertion within a hypermutable C8 tract in exon 11 of *FLCN* was high; further molecular research also confirmed that the poly(C) tract in exon 11 of *FLCN* is a mutation hot spot [12].

Studies of *FLCN* mutations for BHD syndrome are less prevalent in China than they are in Europe and America. Japanese researchers reported gene mutations of *FLCN* in five patients with BHD in 2007; all of the mutations were unique, and four were novel [13]. A recent genetic study of Japanese patients with BHD syndrome published in 2016 included 312 patients from 120 different families and identified 31 *FLCN* sequence variants; two different mutation hot spots, c.1533_1536delGATG in exon 13 and c.1347_1353dupCCACCCT in exon 12, were found [14]. The difference between races may result in a different mutation spectrum in Asian compared with Caucasian populations. Nevertheless, studies of BHD syndrome in Asia are rare. BHD syndrome mutation analysis in Japanese populations concluded that isolated type with pulmonary involvement and recurrent episodes of pneumothoraces were more informative as diagnostic criteria for BHD in the Asian Japanese population [13, 14]. In China, Ren et al. reported sporadic and familial isolated primary spontaneous pneumothorax (PSP) and found that 10 PSP patients had *FLCN* gene mutations, none of whom had other features of BHD [15]. However, further studies of BHD syndrome in Chinese populations are warranted. This study aims to discover the mutation spectrum of the *FLCN* gene in Chinese patients with BHD and attempts to relate the mutation spectrum to the known phenotypes through literature review.

## Methods

### Study population

**Fig. 1:**
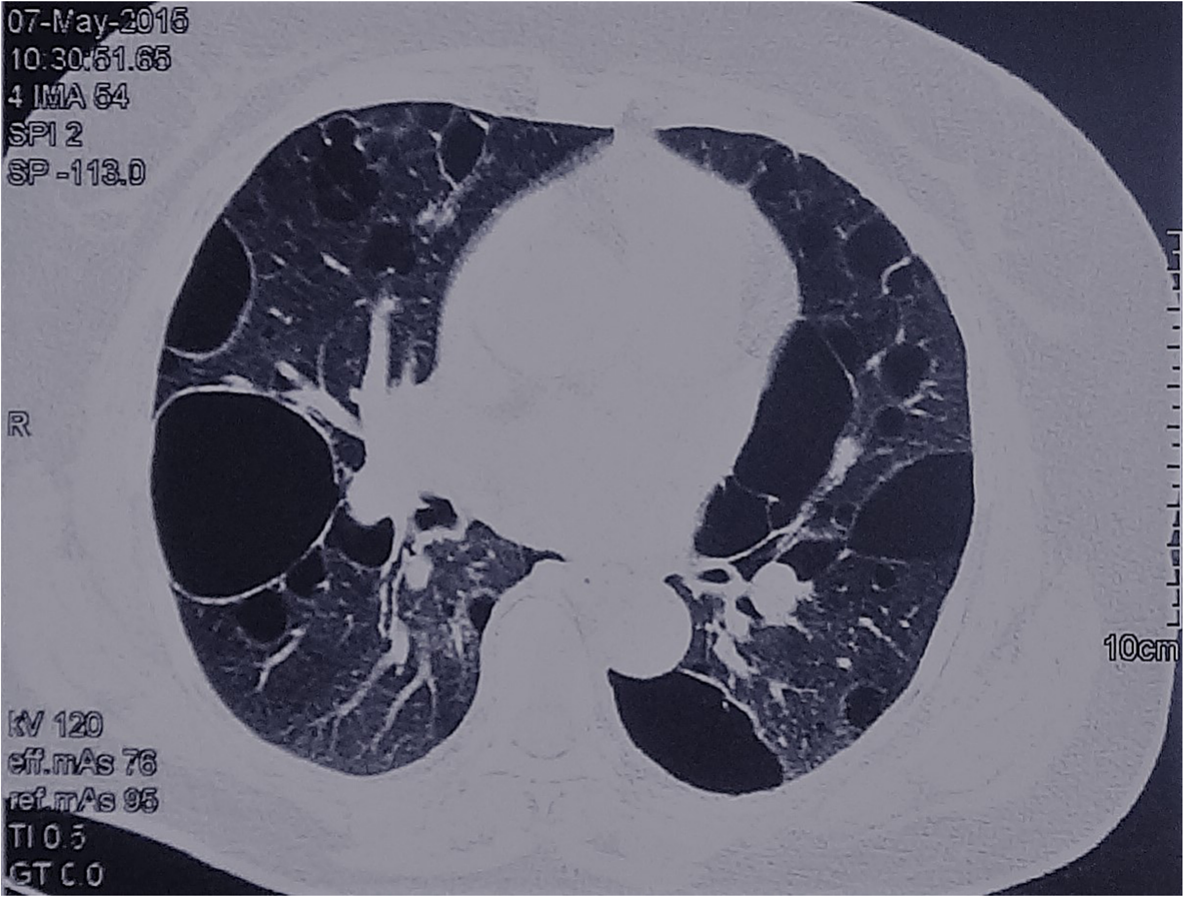
Chest CT showing multiple thin-walled cysts in Patient B24

A total of 51 patients who had been receiving medical care at Peking Union Medical College Hospital (PUMCH) and who had pulmonary cysts in clinical diagnosis from January 2014 to February 2017 were included in the study. The inclusion criteria were BHD symptoms, as described in the review of BHD syndrome diagnosis and management published in *Lancet Oncol*, 2009 [16]. Patients with the following conditions were highly suspected of having BHD: multiple bilateral basally located lung cysts (Fig. 1) with no other apparent cause, a history of episodes of pneumothoraces, first-degree relative with BHD, multiple fibrofolliculomas or trichodiscomas confirmed by dermatologists, probable nephropathy. High-resolution computed tomography (HRCT) results of each patient were assessed by two pulmonary physicians independently. Those with radiology images highly suggestive of other diffuse cystic lung diseases were excluded; these diseases included lymphagioleiomyomatosis, light chain deposition disease, amyloidosis, infectious pneumocystis, tuberous sclerosis, lymphoid interstitial pneumonia and pulmonary Langerhans cell histiocytosis. Patients accompanied by specific symptoms of hereditary syndromes, such as cystic fibrosis, Ehlers-Danlos syndrome, homocystinuria, Marfan syndrome and α1-antitrypsin deficiency, were also excluded from the study [17, 18]. The protocol of this study was approved by the Institutional Review Board committee at PUMCH.

### Mutation analysis of the *FLCN* gene

Genomic DNA from all 51 patients was extracted from peripheral blood leukocytes using Qiagen minibox (according to the manufacturer’s instructions. A total of 14 coding exons with the flanking sequences of the *FLCN* gene were amplified. The software Primier-Primer5 was used to design exon primers. The primer sequences are listed in Additional file 1: Table S1. The amplification reaction mixture (25 μl) was subjected to denaturation at 95 °C for 5 min, followed by 35 cycles at 95 °C for 1 min, annealing at 59-62 °C for 1 min, 72 °C for 3 min, and a final extension at 72 °C for 15 min.

*Sanger* sequencing was carried out on the samples to identify the mutations in each amplicon. Mutations were described according to the recommended nomenclature at http://www.HGVS.org/varnomen [19]. Nucleotide numbers are derived from GenBank accession number NM_144997, assuming that nucleotide 456 is the A of the first ATG translation initiation codon. All mutations were checked in the Human Gene Mutation Database, which is the gold standard resource for comprehensive data on published human inherited disease mutations. Those that had not been previously reported are marked as novel mutations in this article.

Furthermore, for those whose *Sanger* sequencing showed negative mutation results, a multiplex ligation-dependent probe amplification (MLPA) test was applied. MLPA can be used to detect whole-exon deletions and duplications that are not detectable by traditional Sanger sequencing [20, 21].

## Results

### Germline mutation of the *FLCN* gene

**Fig. 2:**
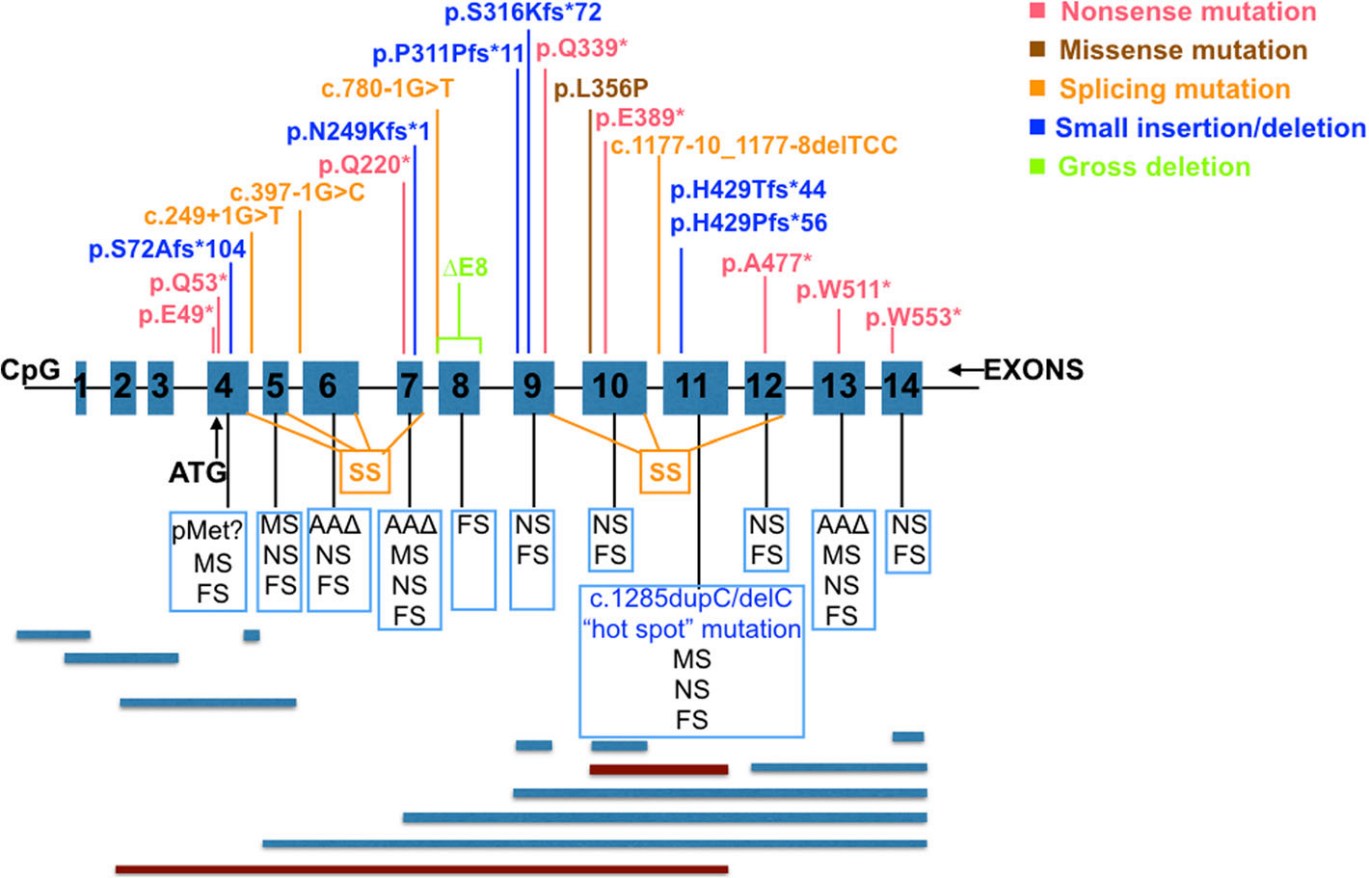
Mutation spectrum of the *FLCN* gene responsible for BHD syndrome. Top of this figure: mutations identified in this study; Bottom of this figure: mutations identified in other studies as reviewed in Schmidt et al. [11], Furuya et al. [14], Zhang et al. [24], Rossing et al. [25]. Definitions of abbreviations: FS = frameshift; MS = missense; NS = nonsense; AAΔ, amino acid deletion inframe; pMet1? = proposed deletion of initiator codon; SS = splice site. ATG = initiator codon. CpG = putative promoter region. △E8 = whole exon 8 loss. Blue bar, intragenic deletion; Brown bar, intragenic duplication

*FLCN* mutations were identified in 27 independent individuals from different families. A total of 20 mutations, with 14 novel and 6 previously known heterozygous *FLCN* mutations (Table 1), were identified in this study. The mutation spectrum of *FLCN* among our patients is illustrated in Fig. 2. Clinical characteristics of these patients are listed in Table 2. The clinical diagnoses of *FLCN* negative patients are available in Additional file 2: Table S2.

**Table 1:** Results of mutation analysis of the *FLCN* gene

Patient	Position (hg19) (NM_144997)	Nucleotide change	Amino acid change	Variant classification (ACMG guideline)	MAF (ExAC, east Asian)
B1	Exon 9 (17122448_449)	c.946_947delAG	p.Ser316Tyrfs*73	pathogenic	absent
B2	Exon 9 (17122462)	c.933delT^a^	p.Pro311Profs*11	pathogenic	absent
B3	Intron 7 (17124943)	c.780-1G > T^a^	Splicing	pathogenic	absent
B4	Exon 7 (17125936)	c.658C > T^a^	p.Gln220*	pathogenic	absent
B5	Intron 5 (17127458)	c.397-1G > C	Splicing	pathogenic	absent
B6	Intron 4 (17131202)	c.249 + 1G > T^a^	Splicing	pathogenic	absent
B7	Exon 4 (17131238)	c.214delA^a^	p.Ser72Alafs*104	pathogenic	absent
B8	Exon 4 (17131295)	c.157C > T^a^	p.Gln53*	pathogenic	absent
B9	Exon 13 (17118304)	c.1533G > A	p.Trp511*	pathogenic	absent
B10	Exon 12 (17118502)	c.1429C > T	p.Arg477*	pathogenic	absent
B11, B12, B13,B19, B20	Exon 11 (17119708)	c.1285dupC	p.His429Profs*27	pathogenic	0.0002388
B14, B15, B24, B27	Exon 11 (17119708)	c.1285delC	p.His429Thrfs*39	pathogenic	0.0002388
B16	Exon 10 (17120492)	c.1067 T > C^a^	p.Leu356Pro	pathogenic	absent
B17	Exon 8 (break points were not determined)	∆E8^a^	p.Trp260Cysfs*12	pathogenic	absent
B18	Exon 9 (17122380)	c.1015C > T^a^	p.Gln339*	pathogenic	absent
B21	Intron 10 (17119825_827)	c.1179-10_1179-8delTCC^a^	Splicing	pathogenic	absent
B22	Exon 10 (17120394)	c.1165G > T^a^	p.Glu389*	pathogenic	absent
B23	Exon 14 (17117051)	c.1658G > A^a^	p.Trp553*	pathogenic	absent
B25	Exon 7 (17129847_836)	c.747_756insGTGATGACAA^a^	p.Asn249Lysfs*1	pathogenic	absent
B26	Exon 4 (17131307)	c.145G > T^a^	p.Glu49*	pathogenic	absent

^a^novel mutations identified in this study

*designates a stop codon

**Table 2:** Clinical manifestations of BHD patients

Patient Number	Sex	Age	Pneumothorax history	Smoking history	Family history	Skin involved	Kidney involved	Lung cysts
B1	F	63	Y	N	N	N	N	Large
B2	M	55	Y	Y	Y	Skin fibrofolliculoma	NA	Medium
B3	F	35	N	N	N	Sarcoma cutis	N	Large
B4	F	46	Y	N	N	N	N	Large
B5	F	41	Y	NA	Y	N	Bilateral renal cysts	Large
B6	F	34	N	N	Y	N	NA	Medium
B7	F	34	Y	N	Y	N	NA	Large
B8	F	54	Y	N	Y	N	N	Large
B9	F	29	Y	N	Y	N	N	Medium
B10	F	57	Y	N	Y	N	NA	Large
B11	F	64	N	N	Y	N	N	Small
B12	F	65	Y	NA	Y	N	N	Large
B13	F	39	Y	N	Y	Skin fibrofolliculoma	NA	Large
B14	M	32	Y	Y	Y	N	N	N
B15	F	50	Y	N	Y	N	N	Small
B16	F	63	Y	N	N	N	N	N
B17	F	50	N	N	Y	N	Bilateral renal cysts	Medium
B18	F	43	Y	N	N	N	N	Large
B19	F	55	N	N	Y	N	Left renal hamartoma	Medium
B20	F	39	Y	N	Y	N	N	Large
B21	F	47	Y	N	Y	N	N	Medium
B22	F	41	Y	N	Y	N	N	Medium
B23	M	44	Y	N	Y	N	N	Medium
B24	F	65	N	N	Y	N	Left renal hamartoma, bilateral renal cysts	Large
B25	F	68	N	N	Y	N	Bilateral renal cysts	Medium
B26	F	58	Y	N	Y	N	N	Medium
B27	F	33	Y	N	Y	N	N	Large

*Definitions of abbreviations*: *F* female, *M* male, *Y* yes, *N* no, *NA* not available

Two novel mutations, c.249 + 1G > T, c.780-1G > T, involving canonical splicing sites, were expected to cause problems in *FLCN* mRNA splicing. One novel mutation, c.1179-10_1179-8delTCC, was predicted to create aberrant splicing of *FLCN* mRNA by Human Splicing Finder. Small deletions involve the following 2 loci: c.933delT, a single bp deletion in exon 9 of the *FLCN* gene leads to a premature termination codon 11 amino acids away from the deletion site, and c.214delA, a single bp deletion in exon 4 of *FLCN* leads to a frameshift mutation that generates a stop codon 104 amino acids downstream. One small insertion in exon 7 was noted: c.747_756insGTGATGACAA, p.Asn249Lysfs*1. Six nonsense mutations, c.157C > T, p.Gln53*; c.658C > T, p.Gln220*; c.1165G > T, p.Glu389*; c.145G > T, p.Glu49*; c.1015C > T, p.Gln339*; and c.1658G > A, p.Trp553* were detected in exons 4, 7, 10, 4, 9 and 14, respectively. One missense mutation, c.1067 T > C, p.Leu356Pro, in exon 10 was discovered.

Among the previously known heterozygous mutations, a single deletion or insertion of cytosine in codon 1285 was identified in nine patients, suggesting that codon 1285 of exon 11 was also a mutation hot spot in Chinese Asians compared with Western Caucasians [22].

**Fig. 3:**
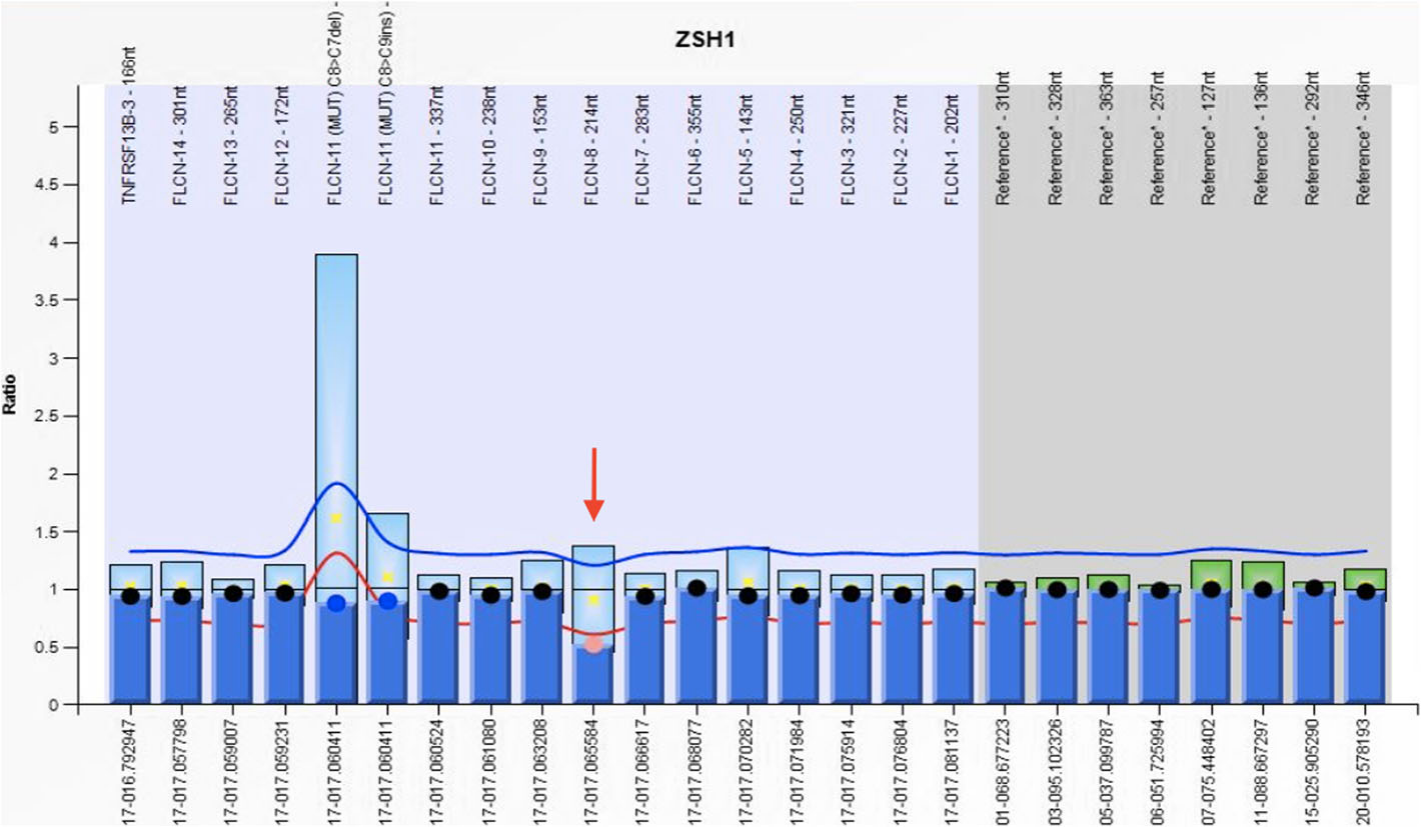
MLPA result of patient B17 showing loss of one copy of exon 8 of *FLCN*

In addition to the point mutations we have found using *Sanger* Sequencing, a whole-exon 8 deletion was detected in one patient using MLPA (Fig. 3).

### Clinical characteristics of these patients

There were 27 independent individuals from different families diagnosed with BHD in our group. Only three patients were male. The median diagnostic age is 48 (29-65 years old). Only seven patients did not have a history of pneumothorax, and two patients had a history of smoking. A total of 22 patients had a family history of pulmonary bulla or pneumothorax. Only three of our patients had skin lesions at the time of diagnosis, and two of them had fibrofolliculoma and one had sarcoma cutis. Renal involvement occurred in 5 out of our 22 patients (five patients were reluctant to do the test), 4 had renal cysts, and two had hamartoma. Considering pulmonary lesions, only two patients did not have CT-detectable pulmonary cysts, but they both had a history of pneumothorax.

## Discussion

When BHD was described in 1977, its incidence was unknown. A study in *Lancet Oncology* in 2009 reported that approximately 200 families had been identified worldwide; but the number of affected families now far outnumbers that report [16]. It was not until 2008 that BHD was recognized by physicians in China [15]. No epidemiological data about the incidence rate of BHD in the Chinese population has been available until now. This is the first report of systematic mutation screening analysis of *FLCN* in a comparably large cohort of Chinese patients with BHD.

In 27 of the 51 patients with symptoms suggestive of BHD, we found 20 mutations in *FLCN*, including 14 novel and 6 already reported mutations. Two novel small deletions of nucleotides, c.933delT and c.214delA, caused a frameshift mutation, resulting in premature termination codons or triggering a nonsense-mediated mRNA decay (NMD). These mutations would generate possible structural and functional changes in folliculin or mRNA degradation; further functional studies were warranted. Three novel mutations involving the splicing sites of exons have been found in this study, c.780-1G > T, c.249 + 1G > T and c.1179-10_1179-8delTCC. The first two could change the acceptor site of intron 7 or the donor site of intron 4, respectively, which would generally cause exon skipping. The third one might create an intronic ESE site of intron 11 as predicted by Human Splicing Finder; however, ESE finder didn’t get the same prediction. Considering the typical clinical manifestations of BHD and also the positive family history (both her father and brother affected) of this patient, the pathogenicity of this variant might be strong. However, further functional tests are needed to validate this prediction. Additionally, six novel nonsense mutations, p.Gln220*; p.Gln53*, p.Gln339*, p.Glu389*, p.Trp553* and p.Glu49*, have been discovered; the resulting premature termination codons will indeed cause truncated protein production and/or NMD. One missense mutation, c1067T > C; p.Leu356Pro, in exon 10 was noted. PolyPhen2, Mutation Taster, FATHMM and PROVEAN gave “probably damaging/ disease causing/ damaging/ deleterious” results, and its REVEL score is 0.841. All the prediction tools supported its pathogenicity. In the future, however, functional tests of this missense variant will be needed to further provide solid evidence. In addition to the identification of single nucleotide variants and intragenic small indels mentioned above, exon deletion was detected by the MLPA test. A whole exon 8 loss was identified in one patient, and a truncated protein or NMD are highly likely to result from the loss of this exon. All of the variants identified in this study were absent from ExAC database except for the recurrent mutation hot spot (c.1285dupC/delC); they were all classified as pathogenic with some manual adjustments such as checkinging PVS1, PM2, PP3 and/or PP4 (http://wintervar.wglab.org/) based on ACMG/AMP 2015 guidelines (Table 1).

Similar to the mutation hot spots reported previously in Caucasians [23], 5 duplication and 4 deletion mutations at c.1285, a hypermutable C8 tract in exon 11, were found in our study, accounting for 33.3% (9/27) of all cases and suggesting that this mutation hot spot does not differ between ethnic populations. No other significant mutations were found to be a potential hot spot in the Chinese population. Nevertheless, 14 of 27 (>50% of total cases) *FLCN* positive patients were reported to have novel mutations, thus demonstrating the diversity of mutation spots along the gene and dramatically broadening the mutation spectrum of *FLCN*. Thus, the data might suggest race differences in the mutation spots between Chinese and Caucasians. However, considering the inactivation role of *FLCN* in the etiology of BHD, it is not surprising to see novel ones spreading all over the genes. More studies with larger populations of Chinese patients with BHD are needed to investigate this topic further.

**Fig. 4:**
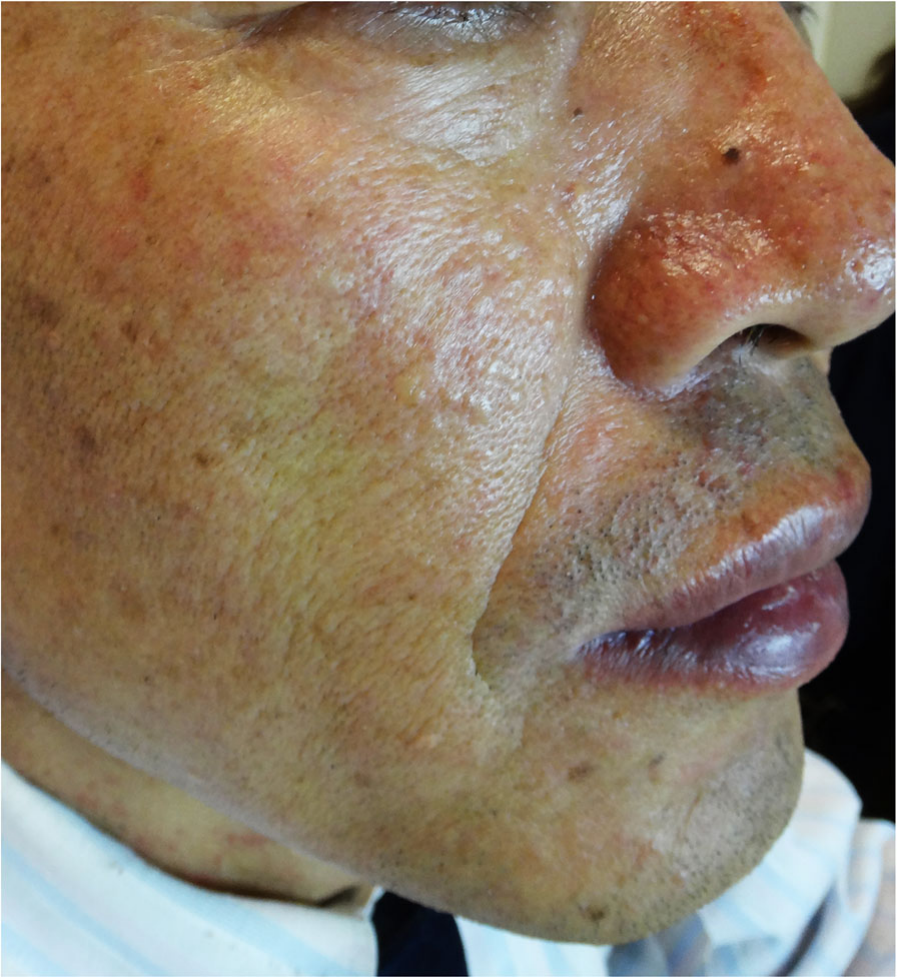
A picture showing Skin fibrofolliculinoma from one patient with BHD syndrome

**Fig. 5:**
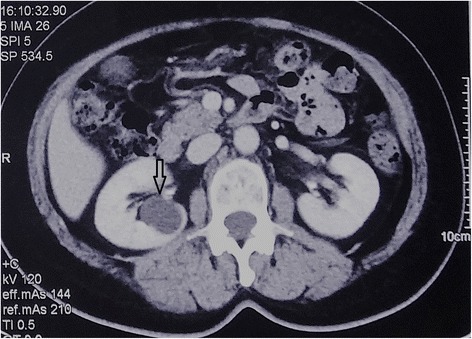
CT image showing renal cyst (arrow) in Patients B24

The first early onset symptom in a substantial proportion of our BHD patients was pneumothorax (74%, 20/27), and a similar clinical pattern was reported in Japanese Asian populations in 2016 [14]. Typical skin fibrofolliculomas were only detected in two patients (Fig. 4); a probable explanation for the low detection rate was that the papules were inconspicuous and asymptomatic. Furthermore, no renal cell carcinoma was diagnosed in our BHD patients, except for 1 with hamartomas and 4 with renal cysts (Fig. 5). Patients enrolled in this study were mostly from a respiratory clinic, which could explain the low renal cell carcinoma rate. Though no significant association between *FLCN* mutation status and lung cyst parameters was stated, Dr. Toro et al. discovered that BHD mutations in exon 9 were associated with more lung cysts than other mutation loci [22]. Nevertheless, that correlation was not identified in our study, and no significant correlations between the extent of clinical manifestations severity and types of gene mutations were noted. Additionally, five of our patients did not undergo renal radiological screening because of the patients’ reluctance. Therefore, we cannot confirm a low prevalence of renal lesions in our group. The small number of patients may also have contributed to a lower rate of renal cancer in this study, although this study has a relatively large sample size compared with other studies of BHD in China.

There are several limitations of our study. Pulmonary cysts exist not only in patients with BHD but also in those with DCLD, such as lymphangioleiomyomatosis and Langerhans cell histiocytosis [5]. *FLCN*, *FBN1*, *COL3A1*, *CBS*, *SERPINA1* and *TSC1*/*TSC2* were involved in different DCLD. Here, we only focused on the *FLCN* gene and BHD syndrome. Zhang et al. developed a new detecting method using a rapid next generation sequencing-based (NGS) panel to differentiate *FLCN* gene mutations in patients with PSP [24]. In future efforts, targeted NGS panel or whole exome sequencing analysis on FLCN-negative cases might be necessary. Nevertheless, here, we provide further genetic characterizations of patients with symptoms of BHD.

## Conclusion

In conclusion, this study reports 14 novel mutations of *FLCN* in 27 patients with BHD and is the first study to demonstrate the mutation spectrum of *FLCN* in a Chinese study population. The mutation spectrum in the Chinese population is even more extensively distributed over the entire *FLCN* gene than that in Caucasians. These genetic findings provide stronger evidence for the clinical molecular diagnosis of BHD in China. Our results suggest that mutation analysis of the *FLCN* gene should be systematically conducted in patients with cystic lung diseases.

## Additional files

Additional file 1: Table S1. Primers of Exon 4 to 14 in the *FLCN* gene. (XLSX 8 kb)

Additional file 2: Table S2. Clinical diagnosis for those patients with *FLCN*-negative results. (XLSX 8 kb)

## Abbreviations

BHD: Birt-Hogg-Dubé syndrome
DCLD: Diffuse cysts lung diseases
*FLCN*: Folliculin
MLPA: Multiplex ligation-dependent probe amplification
mTOR: mammalian target of rapamycin
NGS: Next generation sequencing
NMD: Nonsense-mediated mRNA decay
PUMCH: Peking Union Medical College Hospital
